# Sectoral Differences in Psychosocial Well-Being: The Role of Work Environment Factors Across Public Administration, Healthcare, Pharmaceutical, and Energy Services

**DOI:** 10.3390/bs16010157

**Published:** 2026-01-22

**Authors:** Evija Nagle, Iluta Skrūzkalne, Silva Seņkāne, Otto Andersen, Anna Nyberg, Olga Zamalijeva, Olga Rajevska, Ingūna Griškēviča, Andrejs Ivanovs, Ieva Reine

**Affiliations:** 1 Statistics Unit, Riga Stradiņš University, Dzirciema iela 16, LV-1007 Riga, Latvia; 2 School of Business and Law, Agder University, Gimlemoen 25, 4630 Kristiansand, Norway; 3 Department of Public Health and Caring Sciences, Uppsala University, Husargatan 3, SE-751 22 Uppsala, Sweden; 4 Institute of Psychology, Faculty of Philosophy, Vilnius University, Universiteto g. 9, LT-01513 Vilnius, Lithuania; 5 Department of Health Psychology and Pedagogy, Riga Stradiņš University, Dzirciema iela 16, LV-1007 Riga, Latvia

**Keywords:** autonomy, health risks, job demands–resources model, professional development, sectoral differences, social inclusion, psychosocial well-being, Latvia

## Abstract

The psychosocial well-being of employees is crucial to health and productivity, and it forms the basis for organisational sustainability. Unfortunately, most studies rely on narrow indicators or small samples and thus are not generalisable. The present study aims to identify psychosocial and health-related factors that distinguish employees with high and low SWB and determine whether these effects are universal or sector-specific. A total of 1628 employees with organisations in Latvia’s public administration, healthcare, pharmaceutical and energy sectors participated by completing the Multidimensional Psychosocial Well-Being Scale for Employed Persons (MPSWEP). This instrument assesses five key work environment factors: social inclusion, professional development, work intensity, health risks and autonomy. Subjective well-being (SWB) was measured as a separate outcome variable, and additional self-reported health problems were included as an independent variable in the analysis. Higher odds of high SWB were observed with greater social inclusion (OR = 5.11; *p* < 0.001), whereas higher work intensity (OR = 0.51; *p* < 0.001) and health problems (OR = 0.25; *p* < 0.001) were associated with lower odds of high SWB. Model accuracy was high (AUC = 0.85–0.87), with significant differences between sectors. The results suggest that some resources universally facilitate well-being across sectors, while others exert more sector-specific effects.

## 1. Introduction

Psychosocial well-being is a critical determinant of employee health, performance, and organisational sustainability in the context of a rapidly evolving labour market. Subjective well-being is defined as “an individual’s overall assessment of life satisfaction and emotional experience” (Diener et al., 2018) and is further conceptualised as an employee’s psychological evaluation of their work experience, encompassing both cognitive and affective dimensions (OECD, 2013).

International frameworks emphasise that occupational well-being is multidimensional. The OECD indicates key dimensions in job security, autonomy, skill development, work–life balance, and supportive social relationships (OECD, 2013, 2020). The World Health Organisation also points out that well-being does not mean only the absence of ill health but also includes a safe and supportive environment (WHO, 2022). Complementing these views, the JD-R model explains that when job demands are high, strain increases, but when job resources are sufficient, motivation and resilience improve (Bakker & Demerouti, 2017).

Although research on well-being is extensive, it remains conceptually and methodologically fragmented. Many studies rely on small or context-specific samples, limiting generalisability (Nielsen & Miraglia, 2017), and few provide cross-sector comparisons. Evidence suggests that factors such as autonomy and social support are generally linked to more favourable well-being outcomes, though their relative importance may differ by occupation (Parent-Thirion et al., 2016; Scanlan & Still, 2019). Additionally, substantial variation in study design and sample composition has resulted in inconsistent findings regarding which determinants are universal and which are sector-specific (Nielsen & Miraglia, 2017; Eurofound, 2023).

In response to these limitations, this study adopts a sector-comparative design to assess whether established psychosocial factors demonstrate consistent associations with well-being across different industries. Drawing on the OECD, WHO, and JD-R frameworks, the research investigates well-being among employees in four sectors, aiming to distinguish universal determinants from those influenced by sector-specific conditions.

This study aims to identify the key psychosocial and health-related factors associated with employees’ well-being and to determine whether these associations are consistent across sectors or vary in sector-specific ways.

### Research Questions

RQ1. Which psychosocial and health-related factors are significantly associated with employees’ psychosocial well-being?
RQ2. Which of these associations are consistent across sectors, and which vary in sector-specific ways?

## 2. Conceptualisation and Factors Associated with Psychosocial Well-Being in the Work Environment

### 2.1. Conceptualisation of Psychosocial Well-Being (PSWB)

Psychosocial well-being (PSWB) in the context of the work environment is defined as an employee’s psychological assessment of the quality of their work experience, including both cognitive aspects (e.g., job and life satisfaction) and affective components (e.g., frequency and intensity of positive and negative emotional reactions) (Diener et al., 2018; OECD, 2013). Unlike general quality-of-life studies, SWB in the work environment involves specific resources and requirements that directly determine professional well-being, including social inclusion, autonomy, work intensity, and health risks (Bakker & Demerouti, 2017; WHO, 2022).

The existing scientific literature confirms that the conceptualisation of SWB in the work context has been both fragmented and methodologically inconsistent to date. Some studies have focused on narrow indicators, such as burnout (Maslach & Leiter, 2022) or job satisfaction (Grawitch & Ballard, 2016), which identify only certain negative or one-dimensional aspects but do not capture the complexity of SWB. Other authors have taken a broader approach, including both job resources and demands (Cotton & Hart, 2003; Nielsen & Miraglia, 2017). However, these studies are often based on a limited number of respondents and insufficient sectoral representation, which significantly limits the reliability and generalizability of conclusions. In contrast, international studies with large samples (Eurofound, 2020; Parent-Thirion et al., 2016) provide a broad overview of the quality of working life, but their generalised approach does not allow for a precise determination of which factors are universal and which are specific to sectors (see Table 1).

Analysis of prior research highlights both conceptual and methodological fragmentation in the literature. Approaches that focus solely on individual negative indicators or rely on overly generalised models hinder comprehensive understanding of the multidimensional nature of subjective well-being in the workplace. Consequently, an integrated approach that addresses both positive and negative aspects of well-being and enables broad cross-sector comparisons is required.

The multidimensional MSWEP model used in this study is based on three leading conceptual frameworks: the OECD approach, which integrates cognitive and affective dimensions (OECD, 2013, 2020). The World Health Organisation framework, which emphasises the importance of the work environment in ensuring employee health and psychological safety, and the Job Demands-Resources (JD-R) model, which analyses the balance between demands and available resources (Bakker & Demerouti, 2017). This approach not only deepens theoretical understanding of SWB but also enables the practical identification of universal factors relevant across sectors, as well as sector-specific factors that stand out in specific professional contexts.

### 2.2. Determinants of Psychosocial Well-Being

Research on psychosocial well-being (PSWB) in the workplace is central to identifying key protective factors and risks influencing employee health, motivation, and performance. This focus enhances both theoretical understanding and the development of evidence-based organisational policies and interventions (Bakker & Demerouti, 2017; Helliwell et al., 2021). Previous studies have employed diverse methodologies, including linear regression, binary logistic regression, and structural equation modelling (SEM), yet findings remain inconsistent. These inconsistencies stem from variations in conceptual frameworks, sample sizes, and industry contexts, which limit the generalisability of results (Cotton & Hart, 2003; Eurofound, 2023; Nielsen & Miraglia, 2017; Parent-Thirion et al., 2016). Table 2 summarises the most influential empirical studies on factors associated with subjective well-being.

The studies summarised in Table 2 offer only a partial understanding of the factors influencing employees’ psychosocial well-being (PSWB), primarily due to small sample sizes (Cotton & Hart, 2003; Nielsen & Miraglia, 2017) and reliance on overly generalised international data (Eurofound, 2020; Parent-Thirion et al., 2016). These limitations hinder reliable identification of universal versus sector-specific determinants of subjective well-being. For instance, work intensity and health risks tend to have a greater negative impact in healthcare and education, but are less significant in administrative or technological fields (Eurofound, 2020; Parent-Thirion et al., 2016). This variation suggests that identical factors may yield different outcomes depending on professional context, precluding straightforward generalisation across sectors. This issue underscores a key scientific challenge: while theoretical frameworks (OECD, WHO, JD-R) conceptualise psychosocial well-being as multidimensional, most empirical studies lack an integrated cross-sectoral perspective. Such fragmentation impedes the identification of universal and discipline-specific determinants. To address these gaps, the present study employs a multidimensional conceptual framework and a unified interdisciplinary empirical design, enabling both theoretical refinement and empirical distinction between universal and sector-specific determinants of work-related well-being.

## 3. Research Methodology

### 3.1. Sampling and Data Collection Methods

The research design used a non-probability sampling technique, with convenience sampling in the first stage, and the second stage used target population (census-type) selection in certain sectors. The research targeted Latvian sectors comprising the workforce from four sectors: public administration, healthcare, pharmaceuticals, and energy.

The choice of these sectors is theoretically and practically justified, as the existing literature reveals significant differences in job demands across emotional, cognitive, organisational, and safety aspects. Public administration is a sector characterised by bureaucratic tasks, organisational work, and emotionally demanding work, which are associated with negative emotions, such as exhaustion and job-related tension (Eurofound, 2023). The healthcare sector requires emotionally demanding tasks, high-pressure tasks, and high emotional engagement, which are significantly linked to burnout, a significant aspect concerning the mental health of healthcare professionals (Maslach & Leiter, 2022). The pharmaceutical sector is characterised by stringent regulations, high accuracy, and high cognitive demands, which are significantly associated with high job-related tension (Rehman et al., 2025). Employment within the energy sector is characterised by safety-critical, physically demanding tasks, that is, prolonged working hours, which are significantly linked to high psychosocial tension, a significant aspect concerning fatigability (Amin et al., 2024).

The fact that the study included public, private, and non-profit sector organisations allowed for the evaluation of structural differences in factors such as autonomy, cultural characteristics, resource availability, and job stability, which are acknowledged to affect psychosocial well-being (Bakker & Demerouti, 2017).

Approximately 2000 potential respondents were targeted, resulting in 1628 valid responses and a response rate of 81.6 per cent. Eligibility criteria included being at least 18 years old, actively employed (full-time or part-time), and having sufficient proficiency in Latvian to complete the questionnaire. Data collection was conducted in Latvia from July to September 2024.

### 3.2. Measures

The multidimensional Scale of Psychosocial Well-Being for Employed Persons (MPSWEP) is a newly developed instrument designed to assess key psychosocial aspects of employee well-being in organisational settings. The scale was developed through a multi-stage process that included item generation based on the OECD, WHO, and JD-R frameworks, expert content validation, and two rounds of pilot testing. Exploratory factor analysis supported a six-factor structure representing subjective well-being, social inclusion, professional development, work intensity, health risks, and autonomy (see Table 3).

The factor loadings ranged from 0.60 to 0.88, the KMO was 0.88, and Bartlett’s test was significant (χ^2^ = 11,228, *p* < 0.001), indicating sampling adequacy.

Confirmatory factor analysis further confirmed good model fit (CFI = 0.916; TLI = 0.901; RMSEA = 0.061) (see Table 4).

All the subscales showed good to very good internal consistency, with Cronbach’s alpha ranging from 0.74 to 0.90. The questionnaire also requested demographic and sectoral data, including gender, age, tenure, and employment sector. Age, sex, and tenure were controlled for, as they are known to modify employee well-being outcomes. Subjective health problems were further included, as they are closely related to both working conditions and subjective well-being, and improve model discrimination and calibration, according to Grawitch and Ballard (Grawitch & Ballard, 2016). The health indicator was derived from items of the European Working Conditions Survey and combined two binary symptoms reflecting physical and mental health complaints (Parent-Thirion et al., 2016). The indicator showed adequate internal consistency (Cronbach’s alpha = 0.61) and acceptable agreement for binary indicators (Cohen’s kappa = 0.405, *p* < 0.001; Briggs & Cheek, 1986). Item associations were statistically significant, with chi-square tests indicating a strong relationship (all *p* < 0.001). Based on these findings, a single binary health variable was constructed, coded as presence versus absence of health problems, and used in further analyses. Aggregating physical and mental health symptoms into a composite indicator aligns with recent occupational health research, which conceptualises subjective health as an overall symptom burden (Rhubart et al., 2023; Wienand et al., 2024).

Items were rated on six-point Likert scales, with response options ranging from 1 equals strongly disagree to 6 equals strongly agree for items 1 to 6, and from 1 equals at no time to 6 equals all the time for items 7 to 15 and 23 to 27. Each factor score was calculated as the mean of its items, yielding continuous-scale measures.

### 3.3. Ethical Considerations

The study was approved by the Ethics Committee of Riga Stradiņš University-Decision No. 2-PEK-4/495/2024. The purpose of the study, procedures, data processing principles, and data confidentiality were explained to all participants before they began the questionnaire. Participation was free, anonymous, and voluntary, and all respondents could withdraw from the study at any time without negative consequences.

All data were processed and stored in accordance with the General Data Protection Regulation requirements. The dataset was securely stored on a password-protected computer, accessible only for data analysis purposes to authorised members of the research team. The study was conducted in accordance with the ethical principles of the Declaration of Helsinki, including respect for participant autonomy, confidentiality, and data protection (World Medical Association, 2013).

### 3.4. Data Analysis

Descriptive statistics were means, standard deviations, medians, frequencies, and percentages. Sector differences in working conditions were tested using the Kruskal–Wallis test (including Bonferroni adjustment). Because subjective well-being was not normally distributed and assumptions for linear regression could not be met, it was dichotomised at the median and analysed using binary logistic regression.

The hierarchical binary logistic regression models were estimated by the Enter and Forward Wald procedures. Standard diagnostic tests for model fit and assumptions included likelihood ratio tests, Hosmer–Lemeshow statistics, Nagelkerke R^2^, and assessment of logit linearity. Receiver Operating Characteristic curve analysis and the area under the curve assessed discriminative performance. Odds ratios included 95% CIs, while the level of statistical significance was set at *p* < 0.05.

## 4. Results

### 4.1. Descriptive Statistics

Descriptive statistics were conducted to report on the characteristics of the study participants and are presented in Table 5. As the scales and variables (age, job tenure) were not normally distributed, the Median and Interquartile Range (IQR) were used to describe the data. There were fewer than 5% missing values, so the imputation precision should not bias the results.

### 4.2. Results for RQ1: Which Psychosocial and Health-Related Factors Are Significantly Associated with Employees’ Psychosocial Well-Being?

#### 4.2.1. Sectoral Differences in Psychosocial Work Environment Factors Relevant to Well-Being

To examine potential heterogeneity across employment sectors, Kruskal–Wallis H tests were conducted to compare the distributions of subjective well-being (SWB) and its psychosocial and health-related correlates, including social inclusion, professional development, work intensity, health risks, autonomy, and subjective health problems, across the four employment sectors (see Table 6).

The analysis revealed statistically significant sectoral differences in social inclusion H (3) = 19.54, *p* < 0.001, professional development H (3) = 27.18, *p* < 0.001, work intensity H (3) = 103.92, *p* < 0.001, autonomy, H (3) = 104.82, *p* < 0.001, and health risks H (3) = 32.47, *p* < 0.001. Differences in subjective well-being were also statistically significant, H (3) = 13.69, *p* < 0.001, suggesting variation in employees’ overall well-being across sectors.

Following the omnibus tests, post hoc pairwise comparisons were performed using Bonferroni correction (four groups, six pairwise contrasts; α_adj ≈ 0.0083).

The pattern of differences across sectors indicates that organisational conditions are not uniform. For example, employees in the energy sector reported the highest levels of autonomy and professional development opportunities, whereas healthcare and pharmaceutical employees reported the highest work intensity and exposure to health risks. These results suggest that while certain factors, such as social inclusion and autonomy, have universally positive associations with well-being, others, particularly work intensity and health risks, are sector-specific and context-dependent. Accordingly, subsequent analyses were conducted using sector-adjusted and sector-stratified regression models to explore these relationships in greater depth.

#### 4.2.2. Grouping Strategy for Subjective Well-Being and Application of Binary Logistic Regression

The subjective well-being (SWB) variable was calculated as a composite mean score based on multiple ordinal Likert-type items. As the SWB scale did not satisfy normality assumptions in either the full sample or sector-specific subsamples (W (1627) = 0.991, *p* < 0.001), the measure was dichotomised into low and high well-being using the sample median (4.14). Although median splits are criticised in methodological literature, this approach is nevertheless used in social and clinical research when the aim is to create interpretable group distinctions and support classification-oriented analyses. Median splits are sometimes used to dichotomise continuous or composite ordinal measures, particularly when interpretability and balanced sizes are desirable (DeCoster et al., 2011).

In the present study, the median threshold produced two nearly symmetric, comparably sized groups, thereby supporting model stability and reducing concerns about rare events in binary logistic regression. A binary outcome further facilitates interpretation by allowing a direct assessment of which workplace factors increase the likelihood of belonging to the high well-being group. To ensure analytical robustness, an additional ordinal logistic regression model using the full untreated SWB scale was estimated, yielding a consistent pattern of effects. All cases that rated below the median in subjective well-being were categorised as “low” and all cases that rated above the median as “high” (in this case, ‘0’ = low well-being and ‘1’ = high well-being). Table 7 presents the distribution of SWB groups according to the median-based classification by sector, confirming that there were adequate numbers of respondents in each subgroup for meaningful statistical analysis.

To ensure the validity of the dichotomised grouping and the accuracy of the classification, the optimal cut-off point was determined using Youden’s Index. This index identifies the threshold that maximises both sensitivity and specificity, thereby providing the most accurate distinction between the two groups. The calculation was based on each respondent’s estimated probability of being classified into the respective well-being category in the binary logistic regression model.

Meanwhile, to assess the consistency between the groups obtained using both methods (median method and Youden’s Index threshold), a Pearson Chi-Square test was performed. The results obtained χ^2^ (1) = 88.174, *p* < 0.001; Phi = 0.419, *p* < 0.001 confirmed that there is a statistically significant and moderately strong association between the two classifications. This indicates that the median-based grouping is sufficiently representative and can be used for further regression analysis and data interpretation.

#### 4.2.3. Modelling Approach for Binary Logistic Regression and Validation of Results

To analyse the factors associated with classification into the “high” versus “low” subjective well-being group, a binary logistic regression model was specified as follows: SWB group = Const + X_1_ (social inclusion) + X_2_ (professional development) + X_3_ (health risks) + X_4_ (autonomy) + X_5_ (work intensity) + X_6_ (health problems) + X_7_ (age) + X_8_ (organisational seniority) + X_9_ (gender).

A hierarchical (blockwise) logistic regression was applied: Step 1 included only control variables (age, work experience, and gender); Step 2 incorporated all substantive variables into the model using the Enter method. The omnibus likelihood-ratio test indicated that both models fit significantly better than the intercept-only specification. Explanatory power, indexed by Nagelkerke R^2^, remained limited in the full specifications (Table 8). To ensure methodological transparency, the modelling process and diagnostic checks were performed using both the full sample (N = 1628) and the analytical sample (n = 1323), from which extreme standardised residuals (1.96 < z < −1.96) were removed for the sector-specific analyses.

Because residual-based case removal is not a standard outlier-handling method in logistic regression, we conducted a full set of influence diagnostics on the complete dataset to ensure that none of the removed cases were genuinely influential. Across 1628 observations, all Cook’s distances were below 0.001, the maximum leverage was 0.0236 (far below common influence thresholds), and all DFBETA values were near zero. These results indicate that the excluded cases did not exert undue influence on model estimation. A direct comparison of model performance between the full and reduced samples showed that the direction of the coefficients, the pattern of statistical significance, and the overall classification accuracy remained virtually unchanged. This indicates that the analytic sample (n = 1323) provides a stable and unbiased basis for the sector-level models.

The Hosmer–Lemeshow goodness-of-fit test suggested minor calibration discrepancies for the full sample, which is common in large datasets. To evaluate whether the sample size influenced model calibration, a random subsample of 30% (n = 502) was drawn, and model performance was re-assessed. As shown in Table 9, both models met performance standards; however, a small reduction in explanatory power and AUC was observed due to the smaller sample size.

The suboptimal Hosmer–Lemeshow (*p* < 0.05) values most likely indicate calibration issues rather than poor discrimination. Additional diagnostic procedures were performed in RStudio 4.3.2 to evaluate the model’s classification performance and calibration in the binary logistic regression analysis. The model calibration was checked, as it is not advisable to rely solely on the Hosmer–Lemeshow statistic, especially in large samples. After estimating the final model, the estimated probabilities of belonging to the high SWB group were computed, and a calibration plot was generated using decile clustering.

**Figure 1:**
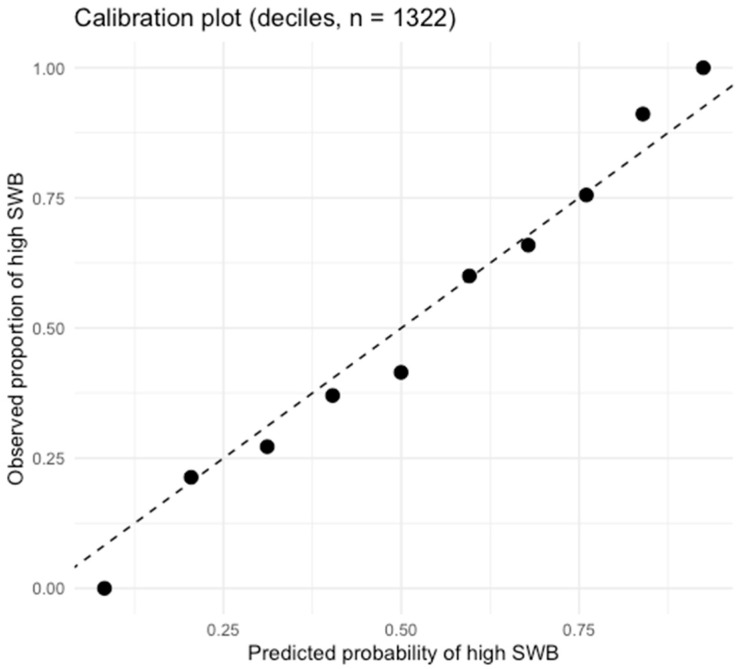
Calibration plot for the binary logistic regression model, showing observed proportions of high subjective well-being across deciles of estimated probabilities.

Calibration analysis was performed on the analytical sample (n = 1322), which was generated by fitting the full model and removing observations with extreme standardised residuals, the same subset of data used for the sectoral analyses. The observed proportion of high SWB was calculated for each decile and plotted against the mean estimated probability (Figure 1).

The resulting calibration plot demonstrated that the model does not systematically overestimate or underestimate the likelihood of high subjective well-being. Thus, despite the Hosmer–Lemeshow test showing a statistically significant result and an expected artefact in larger samples, the graphical and distributional evidence support adequate practical calibration.

The findings for RQ1 indicate that workers’ psychosocial well-being is shaped primarily by three key factors. Social inclusion shows the strongest positive association with well-being, whereas work intensity and self-reported health problems consistently show negative associations, indicating a decrease in well-being. There is a modest positive association with autonomy, whereas professional development does not show a meaningful relationship with well-being. Altogether, RQ1 confirms that psychosocial well-being is determined mainly by the quality of social relations at work and the demands placed on employees.

### 4.3. Results for RQ2: Which of These Associations Are Consistent Across Sectors, and Which Vary in Sector-Specific Ways?

#### Comparative Analysis of Hierarchical Binary Regression Models Associated with Subjective Well-Being

This section presents the results of the hierarchical binary logistic regression analyses, examining which factors are statistically associated with subjective well-being in the full sample and across the four occupational sectors. Two sets of models are demonstrated: (1) full models including the health-risk variable (Table 10), and (2) corresponding models estimated without this variable (Table 11). Comparing these model sets will enable us to gauge the robustness of the findings and assess the extent to which the health-risk indicator impacts overall model performance and substantive conclusions. Although the Hosmer–Lemeshow test indicated a statistically significant lack of fit in the full dataset, the graphical calibration assessment showed no meaningful departures from the ideal reference line, suggesting that the model remains stable and interpretable. When considered together with the consistent AUC values, sensitivity and specificity indices, and stable association patterns across covariates, these results support the adequacy of the model for distinguishing between individuals with high and low levels of subjective well-being.

To guarantee methodological transparency, the modelling process was carried out in a consistent, replicable sequence. First, a full logistic regression model was estimated using the enter method in the complete sample (N = 1628), incorporating all variables included in the analysis. Secondly, extreme cases were identified based on the value of the standardised residuals (|z| < 1.96) and removed to create the analytical sample for sector-specific analyses (n = 1323). Thirdly, the assumption of logit linearity was tested using the Box–Tidwell test. All continuous variables met the linearity assumption, except tenure, which was retained despite being consistently non-significant across all model specifications.

Finally, sector-specific hierarchical models were estimated to use the same modelling strategy. Table 10 and Table 11 present the results of these analyses, comparing models that include the health risk indicator with those that exclude it. Across all sectors, both model specifications demonstrated consistently strong predictive performance, with AUC values between 0.850 and 0.876 and Nagelkerke R^2^ values ranging from 0.49 to 0.69, indicating good overall model fit. The counterintuitive positive associations with SWB, present only in public administration and healthcare (SWB in the total sample OR = 1.28, public administration OR = 1.76, and healthcare OR = 1.28), against the background of non-significant effects in the remaining sectors, suggest that this health-risk indicator does not accurately capture real exposure. This pattern is particularly implausible for public administration, where risks of physical or chemical hazards are minimal. The inconsistency indicates that the two binary items used to operationalise health risks lack sufficient discriminatory precision and could introduce measurement artefacts or suppression effects, thereby distorting substantive interpretation.

Given the fact that the direction, magnitude, and significance of all key variables did not vary as a function of the inclusion of the health-risk measure and there were no meaningful differences in the model-fit indices across models including and excluding this health-risk indicator, Table 10 and Table 11 present the results, interpreted using the models estimated without the health-risk indicator.

The health-risk indicator was included in the initial models based on strong theoretical foundations derived from the JD-R model and international well-being frameworks (WHO, OECD). However, across sector-specific analyses, its effects proved empirically unstable and partly counterintuitive, suggesting limited discriminatory precision rather than a substantive association with subjective well-being. Because including this indicator did not alter the direction or significance of the core predictors, it was excluded from the final sector-specific models to improve clarity, interpretability, and model robustness.

Higher levels of social inclusion (OR = 5.11, *p* < 0.001) and autonomy (OR = 1.17, *p* < 0.05) were associated with increased odds of reporting high subjective well-being in the full sample. By contrast, greater work intensity (Exp = 0.513, *p* < 0.001) and health problems (Exp = 0.245, *p* < 0.001) were associated with reduced odds of high SWB. Most of these patterns were also observable at the sector level, with social inclusion showing the strongest positive association in public administration (Exp = 11.407) and in healthcare (Exp = 5.357). Work intensity demonstrated a consistent negative association with SWB across all industries. Gender reached a significant level only in the health sector (Exp = 0.199, *p* < 0.01); age showed a small but stable positive effect in several sectors.

The health-risk indicator was retained in the models based on sound theoretical underpinnings grounded in the JD-R theory and cross-cultural frameworks of well-being (WHO, OECD). Nonetheless, at the sector level, the effects were empirically inconclusive, with instances of counterintuitive directions, suggesting a lack of precise discrimination, though not a significant relation with perceptions of overall well-being. Since the retention of the health-risk indicator did not impact on the significance of the set predictors, it was removed from the sector-level models to enhance interpretability.

### 4.4. Model Performance and Classification Accuracy

We estimated nested logistic regression models to examine the incremental value of theoretically motivated psychosocial variables beyond basic demographics (age, gender, tenure). The controls-only model showed modest discrimination (AUC = 0.616, 95% CI [0.585, 0.646]; model χ^2^ (3) = 54.085, *p* < 0.001; Nagelkerke R^2^ = 0.062; Hosmer–Lemeshow *p* = 0.567). The full model displayed substantially stronger discrimination (AUC = 0.872, CI [0.854, 0.890]; *p* < 0.001) and explained markedly more variance (Nagelkerke R^2^ = 0.531), though calibration was weaker (Hosmer–Lemeshow *p* = 0.002). Overall, performance improved by Δ AUC ≈ 0.242 and Δ Nagelkerke R^2^ ≈ 0.469, indicating clear incremental validity beyond controls.

Across all sectors, the full models (controls + substantive variables) outperformed the controls-only models. Across the whole sample, accuracy rose from 59.4% to 77.2% (+17.8 percentage points, pp); sector-level gains ranged from +18.7 to +21.4 pp. The largest improvements were in healthcare (sensitivity +39.6 pp), energy services (specificity +30.0 pp), and public administration (sensitivity +22.5 pp, specificity +18.2 pp). Pharmaceutical manufacturing achieved the highest accuracy (76.9%, +21.4 pp) (see Table 12).

Sensitivity analysis was conducted using an ordinal logistic regression (OLR) model estimated in RStudio (clm function, cumulative logit link), to test whether the results depended on the dichotomisation of the SWB scale. This check of robustness was carried out for the full original sample—hence including all extreme cases—rather than for sector-specific subsamples, since sensitivity analyses require sufficient statistical power and are usually conducted at the aggregate level (Thabane et al., 2013). Robustness tests in sub-samples often yield unstable estimates due to sample-size reduction, particularly in ordinal logistic regression, which requires sufficient observations per category. The approach described here is standard in robustness testing and is considered methodologically appropriate for assessing the model’s stability. The full ordered SWB scale was treated as the dependent variable, with the same explanatory variables as in the binary model. Model fit was inspected by pseudo–R^2^ and likelihood-ratio tests, and the proportional-odds assumption was subjected to nominal and scale tests. The results, shown in Table 13, compare odds ratios and significance levels between the main binary logistic regression model (SPSS, N = 1628) and the ordinal logistic regression (RStudio, N = 1628) and indicate a high degree of consistency between the two. Results show that the direction and significance of all main predictors remained consistent across both models. In both approaches, higher social inclusion, greater autonomy, older age, and lower work intensity were strongly and significantly associated with higher subjective well-being. The health problems factor was also robustly and negatively related to SWB, while health risks showed a weaker but consistent positive association in both models. Some differences in odds-ratio magnitudes were observed, especially for variables such as social inclusion and age, which may reflect differences in outcome scaling. This is expected, as large odds ratios in dichotomous models can partly reflect artefacts introduced by categorisation. Overall, this supports the validity of the dichotomised approach for subgroup comparisons while ensuring that overall conclusions are not artefactual or model dependent.

Furthermore, the data analysis revealed a clear structure of factors related to psychosocial well-being, allowing us to distinguish those that operate universally from those that are specific. Overall, the results support our main findings and indicate that social inclusion and work intensity are the main psychosocial factors consistently associated with subjective well-being across all employment sectors. Their effects remained stable in both binary and ordinal models and across industry analyses, highlighting their universal relevance. In contrast, the effects of other variables, such as autonomy, health problems, and demographic characteristics, appear to vary across occupational contexts, highlighting the importance of situational and industry factors in shaping well-being outcomes. Given that the same predictors showed stable, identical effects across both types of models, further industry-specific ordinal analyses were deemed unnecessary.

## 5. Discussion

Psychosocial well-being (PSWB) is a complex and multifaceted phenomenon. In our study, we specifically examined subjective well-being (SWB), a core individual-level component of overall employees’ well-being, and its association with various psychosocial dimensions, including workplace factors, demographic variables, and health-related conditions, across different employment sectors. The identified differences emphasise the importance of analysing well-being in context, accounting for specific working conditions, occupational requirements, and organisational resources. To gain a deeper understanding of these mechanisms, this discussion examines the most important categories identified by the binary logistic regression analysis. Although the median distribution method was used for the dependent variable to improve interpretation, our findings were cross validated using ordinal logistic regression. The consistency of results across both models suggests that the classification choice did not affect the main findings, further strengthening the validity of our interpretations.

### 5.1. Social Inclusion and Employee Subjective Well-Being

The results of the study indicate that social inclusion shows the strongest association with subjective well-being, particularly in public administration. This suggests that employees who feel a sense of belonging and can influence decisions in their work environment are significantly more likely to report higher SWB. This finding aligns with the Job Demands-Resources (JD-R) model, which posits that social resources are pivotal in mitigating stress and fostering emotional well-being (Bakker & Demerouti, 2017). The OECD emphasises that organisations that promote employee engagement and participation also promote higher work efficiency (OECD, 2020). A review by Charalampous et al. found similar results: in remote working conditions, a lack of social connections negatively impacts well-being, whereas the opportunity to actively participate in team processes has a positive impact (Charalampous et al., 2019). Therefore, promoting social inclusion should be considered a strategic priority in all sectors.

### 5.2. Work Intensity and Employee Subjective Well-Being

Across the total sample, higher levels of social inclusion (OR = 5.11, *p* < 0.001) and autonomy (OR = 1.17, *p* < 0.05) were associated with increased odds of reporting high subjective well-being (SWB). In contrast, elevated work intensity (OR = 0.51, *p* < 0.001) and more frequent health problems (OR = 0.25, *p* < 0.001) were associated with a reduced likelihood of high SWB. These associations were largely replicated across sector-specific models. Social inclusion showed the strongest positive association with SWB in both public administration (OR = 11.41) and healthcare (OR = 5.36), while work intensity consistently demonstrated a negative association across sectors. Gender was statistically significant only in the healthcare sector (OR = 0.20, *p* < 0.01), and age exhibited a small but stable positive association in several sectors.

### 5.3. Health Problems and Employee Subjective Well-Being

The presence of health problems was strongly associated with lower subjective well-being across all sectors, with the greatest negative effect observed in the public administration sector. This not only entails that even quite minor physical or psychological symptoms can meaningfully undermine employees’ emotional well-being and day-to-day work experience, but also that such perceptions are closely associated with overall quality of life. These findings are consistent with longitudinal evidence showing that health status is one of the strongest determinants of subjective well-being (Steptoe et al., 2015).

This, however, is a conclusion that must be interpreted with caution considering a methodological limitation of our study: the health-problems indicator is constructed by combining two binary symptoms (physical discomfort and psychological distress). Although recent epidemiological studies often aggregate these symptoms to represent the overall health burden (e.g., Rhubart et al., 2023; Wienand et al., 2024), the composite variable used here provides only a very coarse representation of employees’ health status and may obscure important distinctions among types of health complaints. It is this reduced granularity that limits our ability to determine which physical or mental symptoms exert the strongest influence on well-being outcomes.

Despite this limitation, the uniformly negative relationship between the composite health indicator and subjective well-being underscores the need for health-support mechanisms in organisations. Indeed, future research should use more detailed measures of health, such as symptom scales, clinical indicators, or multi-item health burden indices, to investigate how different physical and psychological health conditions relate to different aspects of employee wellbeing. This aligns with growing research showing that accumulated health symptoms contribute to allostatic load, a key biological pathway linking physical strain to reduced psychosocial well-being (McEwen & Rasgon, 2018).

### 5.4. Age and Employee Subjective Well-Being

Age had a small, yet statistically significant, positive effect on SWB across all sectors. These results suggest that each additional year of life slightly increases the likelihood of higher SWB levels. This trend could be explained by accumulated life experience, better emotional self-regulation, lower stress reactivity, and a more adaptive attitude towards work challenges. Similar findings have been reported by Helliwell et al., who concluded that older workers tend to have higher levels of well-being, partly due to greater emotional stability and life satisfaction (Helliwell et al., 2019). Furthermore, OECD data show that job satisfaction and well-being can increase with age, especially when the work environment is tailored to employees’ ages and provides opportunities for meaningful work (OECD, 2020). Therefore, age plays a small but consistently positive role in contributing to employee well-being. Organisations should consider differences in well-being between age groups, develop intergenerational cooperation initiatives and adapt working conditions to suit different age groups.

### 5.5. Gender and Employee Subjective Well-Being

Gender had an ambiguous, yet statistically significant, impact on the SWB of employed individuals. This impact was particularly significant in public administration and healthcare, where men had significantly lower odds of experiencing high SWB. This result can be partly explained by the uneven gender distribution of respondents—women were significantly overrepresented in the analysed sectors (especially healthcare). This is also consistent with international data: according to an OECD report, women traditionally comprise more than 70–80% of the healthcare workforce (Indicators & Hagvísar, 2019). This imbalance can affect the psychosocial aspects of the work environment, including the quality of social relationships, the availability of support networks, and informal participation in the collective. Men working in predominantly female environments may experience greater isolation, less support from colleagues, and difficulty integrating into the collective identity. Additionally, structural factors need to be considered: men in healthcare more often hold higher, more clinically complex positions (e.g., doctors), which are associated with high levels of responsibility, decision-making pressure, and moral burdens (West et al., 2016). These factors can significantly impact their emotional balance and SWB. At the same time, gender-specific expressions and perceptions of well-being may differ. Research shows that women emphasise emotional well-being more often and are more inclined to talk about psychological discomfort, whereas men tend to internalise stress or express it in somatic form (Batz-Barbarich et al., 2018). Therefore, lower well-being in the male group may not always reflect the actual situation, but rather different perceptions of well-being and ways of expressing it.

Overall, these results confirm that gender is not merely a demographic control variable, but a significant psychosocial determinant. Therefore, employers, particularly those in sectors with pronounced gender imbalances, should develop inclusive, gender-sensitive organisational policies that promote equal opportunities and emotional well-being, assess gender-specific needs and establish support mechanisms in professional environments.

### 5.6. Professional Autonomy and Employee Subjective Well-Being

In this study, autonomy showed a significant positive association with subjective well-being (SWB) in both the overall sample and the energy sector. This indicates that employees who have greater freedom to organise their work, make independent decisions, and control task execution tend to experience higher well-being. The particularly strong effect observed in the energy sector may reflect the importance of individual decision-making and professional competence in technically complex and safety-sensitive environments, where autonomy enhances both efficiency and intrinsic motivation. According to Deci and Ryan’s Self-Determination Theory (SDT), autonomy represents one of the three basic psychological needs that foster intrinsic motivation, personal growth, and emotional well-being (Deci & Ryan, 2008). When employees perceive genuine influence over their work and decision-making processes, they are more likely to experience psychological fulfilment and sustained engagement. Conversely, environments with excessive external control or limited flexibility can undermine motivation and psychological resilience. The absence of a significant effect in other sectors, particularly healthcare, likely reflects structural and procedural constraints. Healthcare work is highly standardised, governed by strict hierarchies, clinical guidelines, and regulatory frameworks that limit professionals’ ability to adapt decisions to specific situations or patient needs. This constrained autonomy may diminish intrinsic motivation and contribute to emotional exhaustion over time. Indeed, previous studies suggest that limited decision latitude in healthcare is linked to higher levels of burnout, moral distress, and reduced well-being (Cerela-Boltunova et al., 2024; Nagle et al., 2023a, 2023b, 2024; West et al., 2018). These findings underscore that professional autonomy is not a universally available resource but is shaped by sectoral structures and organisational design. Promoting autonomy through participatory decision-making, decentralised management, and flexible work organisation could be a key intervention strategy for improving well-being, particularly in rigid or hierarchically organised systems such as healthcare. Efforts to enhance professional discretion should, however, balance employee empowerment with patient safety and quality-of-care requirements.

Overall, the results confirm that subjective well-being (SWB) is a complex, context-sensitive construct shaped by the interplay among job resources, psychosocial factors, and organisational culture. Strengthening professional autonomy, together with social inclusion and supportive leadership, can substantially enhance well-being and resilience across occupational sectors.

### 5.7. Health Risks and Employee Subjective Well-Being

The health risk indicator was theoretically sound; however, its relationship with subjective well-being in sectoral analyses proved to be empirically unstable and partly contradictory. Consequently, this variable was excluded from the final sectoral models to enhance the clarity and interpretability of the results.

### 5.8. Work Experience and Employee Subjective Well-Being

Work experience did not have a statistically significant direct effect on well-being, but several indirect patterns were observed. A longer tenure was often associated with higher social inclusion and perceived autonomy, suggesting that organisational familiarity and accumulated competence may indirectly enhance well-being. Conversely, early-career employees may experience greater insecurity and emotional strain. Similar trends have been noted in OECD data, where job stability and familiarity with organisational culture are associated with higher satisfaction and engagement (OECD, 2020). Therefore, although work experience may not serve as a primary determinant, it can moderate the relationship between job resources and well-being, suggesting that tailored support for newly employed workers could yield long-term benefits.

Overall, the study’s findings confirm that subjective well-being is a multidimensional, context-sensitive construct shaped by the interaction among job resources, psychosocial factors, and organisational culture. Strengthening social inclusion, professional autonomy, and health support mechanisms can substantially enhance employee well-being and resilience across occupational sectors.

## 6. Theoretical and Practical Implications

### 6.1. Theoretical Implications

The results of this study contribute to the theoretical understanding of psychosocial well-being (PSWB) in organisational contexts on several grounds. First, the consistent significance of social inclusion and work intensity across all four sectors provides strong empirical support for the JD-R model, which stipulates that job resources enhance well-being while job demands undermine it (Bakker & Demerouti, 2017). Our findings extend this theoretical framework by showing that only two factors, namely, social inclusion as a core resource and work intensity as a central demand, act as universal determinants of well-being regardless of sector-specific structures, work cultures, or occupational risk profiles. This refines previous research, which has often identified a broader set of factors associated with well-being but has not systematically examined whether these associations hold consistently across different industries (Parent-Thirion et al., 2016; Charalampous et al., 2019). Second, the present study contributes to multidimensional well-being theories by integrating three conceptual frameworks: OECD well-being principles, the WHO healthy workplace guidelines, and the JD-R model, by empirically validating such integration. While existing research generally operationalises workplace well-being through narrow indicators, such as burnout or job satisfaction (e.g., Maslach & Leiter, 2022; Grawitch & Ballard, 2016), the current study provides comprehensive evidence that psychosocial well-being is contingently shaped by the configuration of both structural demands and relational resources. This emphasises the need to conceptualise PSWB as an inherently context-dependent construct, inextricably embedded within organisational characteristics rather than solely an individual psychological state. Third, the results for autonomy, health-related factors, age, and gender support both the assumptions of sectoral contingency theories and social exchange theory (SET). The fact that autonomy was related to well-being only within specific sectors confirms that job resources act differently across organisational structures, regulatory constraints, and operational risks (Deci & Ryan, 2008; Bakker & Demerouti, 2017). Similarly, the negative effect from health problems agrees with research into allostatic load-the idea that well-being is reduced regardless of job context, due to an accumulation of physical and psychological strain (McEwen & Rasgon, 2018). From these findings, it can be concluded that well-being arises from an interplay between structural conditions and the individual’s health, rather than solely from job characteristics. Fourth, the study contributes to the emerging literature on sector-specific well-being mechanisms, a domain in which empirical evidence remains limited. Previous research has largely focused on single-sector analyses, such as healthcare or education, offering little insight into how the key factors associated with well-being may differ across industries (Nielsen & Miraglia, 2017; Scanlan & Still, 2019). By employing a sector-comparative analytical design, this study shows that while some well-being determinants are general, others demonstrate significant sectoral variation, such as autonomy and health-related factors. This nuanced understanding questions the generalisability assumption often implicit in cross-sectional studies and underscores the need for theoretical models that explicitly account for variation at the industry level.

Cumulatively, these findings enhance theoretical insights by identifying which psychosocial mechanisms are universal and which are contextually bound; developing multidimensional well-being models with empirical data from diverse occupational settings; and highlighting how organisational structures shape the relationships among job resources, job demands, and employee well-being.

### 6.2. Practical Implications

These insights have some direct practical implications. Because social inclusion and work intensity serve a universal purpose, all types of organisations should focus on strengthening social connectedness, communication quality, participatory decision-making, and manageable workload structures. Interventions such as participatory leadership, team-based problem-solving, and work redesign can systematically improve well-being by reinforcing core job resources and reducing excessive demands (Bakker & Demerouti, 2017). At the same time, strategies need to be sector-specific, as there is contextual variation in job structures and risk profiles across sectors. In healthcare, interventions need to target emotional strain, opportunities for recovery, and resilience-building practices. Public administration calls for initiatives to relieve bureaucratic overload and for transparent, collaborative communication. Pharmaceutical manufacturing needs more balanced cognitive demands, clearer processes, and better workflow organisation. The energy sector, characterised by both safety-critical tasks and high levels of autonomy, calls for continuous investment in operational safety and competence development, as well as in decision-making flexibility. Altogether, the research provided evidence-based guidance for designing both universal and sector-specific organisational policies that improve social inclusion, balance demands with adequate resources, strengthen professional autonomy where possible, and offer health-support mechanisms. These will significantly improve employees’ psychosocial well-being and organisational functioning.

## 7. Limitations

Furthermore, several limitations should be considered when interpreting these findings. While self-assessment questionnaires offer an opportunity to capture employees’ subjective experiences, they may be subject to perception bias and societal expectations. While the study covers four important sectors of employment in Latvia, the sample is not fully representative of the total labour market. This provides clear analytical contrasts between sectors but limits broader generalisability. The study was based on a non-probability sample, and some sectors—most notably healthcare—displayed a pronounced gender imbalance, with women substantially overrepresented. This asymmetry may limit the generalisability of gender-related findings and reduce the precision of estimates for male respondents. Furthermore, unbalanced gender distributions reduce statistical power for detecting interaction effects, particularly in sector-stratified analyses.

Furthermore, the composite measure of subjective health problems comprises only two binary symptoms and provides a crude representation of employees’ health status, potentially masking differences between physical and psychological complaints. These modelling limitations do not affect the substantive conclusions but imply that more detailed health indicators and sector-specific analytical designs are required in future studies. Although the modelling approach used in this study produced robust and stable estimates, several methodological limitations must be acknowledged. Firstly, when interpreting the results, it is important to acknowledge that several odds ratios, particularly those substantially above 1, may appear large. Given that subjective well-being (SWB) was operationalised as a dichotomous variable via median split, these effect sizes should be interpreted with caution. Large odds ratios in logistic regression may, in part, reflect artefacts introduced by categorising continuous or ordinal variables, rather than indicating true underlying effect magnitudes. This issue has been noted in methodological literature, which highlights that dichotomisation can exaggerate group differences, reduce variability, and distort the shape of relationships (Royston et al., 2006; Streiner, 2002). Nevertheless, the decision to dichotomise SWB in this study was motivated primarily by interpretational clarity and the practical aim of identifying high-risk and high-resource groups in applied settings. Importantly, robustness checks using ordinal logistic regression produced substantively similar patterns, lending support to the reliability of the findings despite.

Second, model calibration using the Hosmer–Lemeshow test indicated small deviations in the fit of the full-sample model, which may reflect sectoral heterogeneity or large-sample sensitivity. To further investigate this, we performed additional validation procedures, including ROC-based discrimination, AUC confidence intervals, calibration plots, and 30% random subsample analysis. These tests demonstrated consistent model performance and acceptable calibration, suggesting that the HL test result should be interpreted with caution and not as the sole evidence of non-compliance.

A further methodological limitation concerns the exploratory removal of cases with large, standardised residuals when preparing the analytical sample for the sector-specific models. Residual-based filtering is not a standard outlier-management strategy in logistic regression and may, in principle, reduce generalisability. However, influence diagnostics conducted on the full sample indicated that none of these observations were influential in terms of parameter estimation: all Cook’s distance values were below 0.001, the maximum leverage (hat) value was 0.0236, well below commonly cited influence thresholds (h > 0.05) and all DFBETAs were near zero. Moreover, comparisons across model specifications showed that coefficient directions, effect magnitudes, and overall model performance (Nagelkerke R^2^, AUC, and fit indices) remained virtually unchanged regardless of whether these cases were retained or removed. This suggests that excluding high-residual cases did not bias the substantive conclusions. Influence-based diagnostics (e.g., Cook’s distance, leverage, DFBETAs) are more appropriate for identifying potentially influential observations and are therefore recommended for future research. Residual-based screening should be used only as an exploratory tool, not as a primary criterion for case exclusion. Finally, although most variables showed the theoretically expected associations, the counterintuitive positive relationship between health risks and subjective well-being, observed only in a few sectors, led to the exclusion of this variable from the final sectoral models. Its removal did not change the direction or significance of the remaining associations, indicating that the overall model structure is robust.

## 8. Conclusions

This study aimed to identify the factors associated with employees’ subjective well-being (SWB) and to compare their relative importance across employment sectors using binary logistic regression. The findings indicate that the mechanisms linked to well-being are not universal but vary across sectors, reflecting differences in structural conditions, workload intensity, and organisational culture. Across the full sample, social inclusion and lower work intensity emerged as the only universal determinants of higher well-being, whereas all other factors operated sector-specifically. Although social inclusion, autonomy, and health-related indicators were consistently associated with well-being, the strength and direction of these associations varied across sectors.

### 8.1. Public Administration Sector

Well-being was primarily associated with higher levels of social inclusion and better health status, highlighting the importance of social resources and health protection measures in maintaining employee well-being.

### 8.2. Energy Sector

Higher professional autonomy was significantly related to better well-being outcomes, whereas increased work intensity was associated with lower well-being. These findings suggest that decision latitude and workload balance are essential to sustainable well-being in this sector.

### 8.3. Pharmaceutical Sector

Elevated work demands and self-reported health problems were significantly associated with lower well-being. This pattern indicates that both psychosocial strain and physical risk exposure may contribute to reduced well-being among employees in this field.

### 8.4. Healthcare Sector

The analysis revealed that gender and job demands were among the significant factors associated with lower well-being. These findings point to the potential risk of burnout in environments characterised by high emotional and cognitive load combined with gender imbalance.

Across all sectors, the study underscores the importance of creating inclusive, supportive, and resource-rich work environments that address both psychosocial and health-related needs.

## 9. Future Research

The results of this study indicate that the factors associated with psychosocial well-being (SWB) differ across sectors due to variations in structural characteristics, occupational conditions, and organisational culture. Since well-being is a context-dependent construct, future research should prioritise sector-specific analyses rather than applying a universal approach to all employee groups. Attention should be given to professions where reduced well-being has been identified and where work is performed under conditions of high emotional, cognitive, and physical demands, limited professional autonomy, and high moral responsibility. The healthcare sector demonstrates critically low well-being indicators, with chronic stress and moral strain contributing to emotional exhaustion and reduced system stability (West et al., 2018; Nagle et al., 2023b). Future research could apply weight adjustments within the healthcare sector to correct gender disproportionality, thereby improving the accuracy of gender-specific estimates and reducing potential sampling bias.

Future studies should adjust for subjective health to reduce confounding, as subjective health problems are associated with both perceived working conditions (e.g., coping with demands, autonomy) and well-being. Including a health indicator in future models can help mitigate omitted-variable bias and more accurately identify the direct association between working conditions and SWB. Furthermore, it is important to distinguish between direct and indirect pathways to determine whether working conditions influence SWB through health problems. This can be achieved by estimating parallel models (with and without health indicators) and, where feasible, using formal mediation analyses such as counterfactual mediation or structural equation modelling (SEM) to distinguish total, direct, and indirect effects. To ensure the validity and generalisability of future findings, sensitivity and robustness analyses are recommended. Researchers should examine how the inclusion of health indicators influences effect sizes and discrimination metrics (e.g., AUC) and evaluate the robustness of results across different occupational sectors through interaction terms or stratified models. In addition, future research should consider individual-level determinants such as personality traits, psychosocial risk factors, institutional support mechanisms, and organisational culture. Employing longitudinal and mixed-method designs would allow researchers to capture the dynamic nature of well-being and identify causal relationships between working conditions, health, and SWB.

## Figures and Tables

**Table 1:** Conceptualization of Work-Related Psychosocial Well-Being (PSWB).

Author, Year	Factors Included	Sector(s)	Main Limitations
Maslach and Leiter (2022)	Burnout (emotional exhaustion, depersonalization), professional efficacy	Healthcare, education	Focusing only on negative aspects
Grawitch and Ballard (2016)	Job satisfaction, stress level	Administration	One-dimensional approach
Cotton and Hart (2003)	Job demands and resources	Various sectors	Small samples, no cross-sector comparisons
Nielsen and Miraglia (2017)	Job satisfaction, engagement, psychological stress	Social services	Lacks cross-sectoral analysis
Parent-Thirion et al. (2016)	Work quality indicators (work–life balance, social support)	Multiple EU sectors	Large sample, but findings overly generalised; SWB not conceptualised as multidimensional
Eurofound (2023)	Quality of working life, social inclusion	Multiple EU sectors	Very broad comparisons; no sector-specific analysis; SWB not directly defined as a construct

**Table 2:** Empirical studies examining psychosocial work factors and employee well-being.

Author, Year	Method	Key Factors	Results (Main Findings)	Sector/Sample
Cotton and Hart (2003)	Linear regression	Social support, autonomy, job demands	Social support and autonomy significant across all sectors; job demands important only in certain professions	Australia, various sectors
Nielsen and Miraglia (2017)	SEM	Social inclusion, health risks	Social inclusion is found to be a universal factor; health risks significant mainly in healthcare	Scandinavian countries
Parent-Thirion et al. (2016)	Regression models (EWCS data)	Work–life balance, social support, job demands	Work–life balance and social support showed stable associations with SWB, whereas job demands varied substantially across sectors	Europe, large-scale samples
Eurofound (2021)	Regression analysis	Professional development, working conditions	Professional development associated with higher SWB; effect sizes varied by sector	European employees

**Table 3:** Principal Components Analysis with Varimax Rotation, 23 Items.

Component Rotation Matrix							
Number of Items	F1	F2	F3	F4	F5	F6	h^2^
Item 21	0.86						0.77
Item 20	0.81						0.75
Item 22	0.81						0.69
Item 19	0.80						0.69
Item 23	0.76						0.65
Item 2	0.66						0.59
Item 7	0.60						0.51
Item 4		0.75					0.66
Item 3		0.74					0.65
Item 5		0.70					0.60
Item 6		0.67					0.58
Item 1		0.64					0.52
Item 15		0.60					0.45
Item 18			0.86				0.77
Item 17			0.83				0.74
Item 16			0.76				0.60
Item 14				0.85			0.73
Item 10				0.64			0.70
Item 11				0.64			0.65
Item 8					0.88		0.82
Item 9					0.86		0.80
Item 13						0.86	0.79
Item 12						0.84	0.79
Initial Eigenvalues	4.62	3.40	2.08	1.82	1.82	1.70	
% of Variance	20.09	14.77	9.08	7.95	7.90	7.41	67.21

Note. The table shows factor weights above 0.4 Factors were obtained using Exploratory Factor Analysis (EFA) in SPSS 31, applying Principal Component Analysis (PCA) with Varimax rotation and Kaiser normalisation to optimise the factor structure. F1—Subjective well-being, F2—Social inclusion, F3—Professional development, F4—Work Intensity, F5—Health risks, F6—Autonomy. The full item set and complete survey version of the MPSWEP scale are provided in Supplementary Materials.

**Table 4:** Confirmatory Factor Analysis: 6 Factors, 23 Items.

Factor	Item	Estimate	SE	*p*	95% CI Lower	95% CI Upper
1	Item 22	0.862	0.024	<0.001	0.812	0.908
	Item 21	0.826	0.019	<0.001	0.787	0.865
	Item 20	0.797	0.021	<0.001	0.755	0.839
	Item 23	0.781	0.022	<0.001	0.738	0.825
	Item 19	0.748	0.019	<0.001	0.712	0.787
	Item 7	0.723	0.026	<0.001	0.671	0.776
	Item 2	0.711	0.024	<0.001	0.662	0.758
2	Item 4	0.874	0.028	<0.001	0.817	0.931
	Item 3	0.863	0.027	<0.001	0.809	0.918
	Item 5	0.851	0.029	<0.001	0.792	0.909
	Item 6	0.713	0.027	<0.001	0.661	0.766
	Item 15	0.692	0.030	<0.001	0.632	0.752
	Item 1	0.679	0.029	<0.001	0.622	0.736
3	Item 17	1.948	0.062	<0.001	1.826	2.071
	Item 18	1.901	0.058	<0.001	1.787	2.015
	Item 16	1.313	0.060	<0.001	1.194	1.431
4	Item 10	0.957	0.028	<0.001	0.902	1.011
	Item 11	0.898	0.029	<0.001	0.839	0.956
	Item 14	0.559	0.030	<0.001	0.500	0.618
5	Item 8	1.315	0.045	<0.001	1.226	1.404
	Item 9	1.213	0.040	<0.001	1.134	1.291
6	Item 12	1.117	0.041	<0.001	1.035	1.199
	Item 13	0.966	0.039	<0.001	0.893	1.043

Notes. The table presents factor loadings above 0.3. Factors were identified using Confirmatory Factor Analysis (CFA) in Jamovi 2.7.6, applying Maximum Likelihood Estimation (ML) with robust standard errors to validate the factor structure. F1—Subjective well-being, F2—Social inclusion, F3—Professional development, F4—Work Intensity, F5—Health risks, F6—Autonomy.

**Table 5:** Demographic characteristics of respondents by sector.

Variable	Total (N = 1628)	Public Administration (n = 280)	Healthcare (n = 419)	Pharmaceutical Manufacturing (n = 441)	Energy Services (n = 488)
Gender (%)					
Women (N, %)	1053 (64.7)	228 (81.1)	389 (92.4)	285 (64.6)	151 (30.9)
Men (N, %)	575 (35.3)	52 (18.5)	30 (7.1)	156 (35.4)	337 (69.1)
Age, years					
Median	46	51.5	49	41	46
IQR (75–25)	63	63	60	48	45
Mean (SD)	45.89 (11.9)	50.03 (12.4)	47.1 (12.4)	42 (12.2)	45.2 (9.7)
Tenure, years (in the company)					
Median	13.46	10	8	6	17.13
IQR (75–25)	56	54	56	48	49
Mean (SD)	13.46 (11.9)	14.1 (12.8)	12.7 (12.7)	9 (9.6)	17 (11.5)

Notes. N denotes the total sample size; n denotes sector-specific subsample sizes.

**Table 6:** Differences between employment sectors in key study variables.

Factors	Sig	Sig. Diff Between Sectors (*p* < 0.05)
Social inclusion	19.54 (3) ***	State-Energy Pharma-Energy Hospital-Energy
Work intensity	103.923(3) ***	All
Autonomy	104.818(3) ***	All (except Energy-State)
Subjective well-being	13.685(3) ***	Pharma-Energy

Note. *** indicates statistical significance at *p* < 0.05. Results are based on the Kruskal–Wallis test (n = 1628, listwise). Significance levels were adjusted using Bonferroni correction (cut-off = 0.05 for the type of company).

**Table 7:** Type of Company by Subjective Well-Being Groups.

Type of Company	Low Well-Being N (%)	High Well-Being N (%)	Total (N)
Public administration	142 (17.8)	138 (16.7)	280
Healthcare	203 (25.4)	218 (26.4)	421
Energy	209 (26.1)	279 (33.7)	488
Pharmacy	247 (30.8)	192 (23.2)	439
Total	801 (100)	827 (100)	1628

Notes. N = number of respondents; % = percentage within each company type. N = 1628.

**Table 8:** Results of Binary Logistic Regression for Subjective Well-Being (SWB): Comparison of the Full Model and the Control-Variable-Only Model.

	1 Model						2. Model					
	Full Model (n = 1628)			Control Model (n = 1628)			Full Model (n = 1323)			Control Model (n = 1323)		
Variable’s	OR	Sig	CI 95% [Lower Upper]	OR	Sig	CI 95% [Lower Upper]	OR	Sig	CI 95% [Lower Upper]	OR	Sig	CI 95% [Lower Upper]
Age (years)	1.03	<0.001	[1.01; 1.04]	1.03	0	[1.02; 1.04]	1.04	<0.001	[1.03; 1.06]	1.03	0	[1.02; 1.05]
Work experience (years)	1.00	0.541	[0.99; 1.01]	1.00	0.89	[0.98; 1.01]	1.01	0.109	[0.99; 1.03]	1.00	0.64	[0.99; 1.01]
Gender (men)	0.87	0.332	[0.66; 1.14]	1.10	0.39	[0.88; 1.38]	0.81	0.205	[0.59; 1.11]	1.13	0.3	[0.89; 1.42]
Social inclusion	2.78	<0.001	[2.32; 3.33]				5.36	<0.001	[4.22; 6.81]			
Professional growth	1.03	0.283	[0.97; 1.10]				1.02	0.468	[0.95; 1.11]			
Work intensity	0.58	<0.001	[0.49; 0.69]				0.45	<0.001	[0.37; 0.54]			
Health risks at work	1.17	0.002	[1.05; 1.29]				1.28	<0.001	[1.14; 1.44]			
Autonomy	1.11	0.087	[0.98; 1.26]				1.24	0.003	[1.07; 1.43]			
Health problems	0.37	<0.001	[0.28; 0.49]				0.24	<0.001	[0.18; 0.33]			
Nagelkerke R^2^	0.379			0.044			0.542			0.062		
Hosmer–Lemeshow	<0.001			0.706			<0.001			0.567		
Cut-off	74.5			58.1			78.5			59.4		

Note. OR = odds ratio; *p* = probability value; 95% CI = 95% confidence interval.

**Table 9:** Model performance indicators for binary logistic regression assessing associations with Subjective Well-Being (SWB).

Model	Sample	Nagelkerke R^2^	Hosmer–Lemeshow	AUC	χ^2^
1.	Entire sample N = 1628 (SPSS)	0.323	0.001	0.787; *p* < 0.001 (CI 95% 0.765–0.802)	418.884 (3; 1511) ***
2.	Random sampling (30% of the entire sample, N = 502) SPSS	0.266	0.223	0.764; *p* < 0.001 (CI 95% 0.723–0.805)	107.030 (2) ***

Note. R^2^ = Nagelkerke coefficient of determination; AUC = area under the curve; χ^2^ = chi-square test statistic; *p* = probability value; 95% CI = 95% confidence interval. *** *p* < 0.001.

**Table 10:** Comparison of the impact of various factors on Subjective Well-Being (SWB), as determined by hierarchical binary regression models in different sectors.

	Exp(b)				
Variable’s	Entire Sample N = 1323	Type1 (Public Administration) N = 217	Type2 (Healthcare) N = 325	Type3 (Energy) N = 432	Type4 (Pharma) N = 349
Social inclusion	5.426 ***	13.574 ***	6.378 ***	4.433 ***	4.356 ***
−Work intensity	0.451 ***	0.265 ***	0.389 **	0.465 ***	0.424 ***
Health risks	1284 ***	1761 *	1284 ***		
−Health problems	0.249 ***	0.078 ***	0.338 ***	0.232 ***	0.374 ***
Autonomy	1.170 *			1.440 **	
−Age	1.047 **	1.036	1.040 *	1.071 ***	1.053 ***
Tenure					
Gender (men)		0.209 *	0.272 *		
Nagelkerke R Square	0.542	0.702	0.547	0.547	0.490
Hosmer–Lemeshow test *p*	0.001	0.753	0.263	0.153	0.071
AUC	0.876 ***	0.863 ***	0.851 ***	0.866 ***	0.866 ***

Notes. Exp(b) denotes odds ratios obtained from hierarchical binary logistic regression models. A minus sign (−) before a variable indicates that higher values of this factor decrease the likelihood of high subjective well-being (Exp(b) < 1), whereas values of Exp(b) > 1 indicate an increased likelihood of high subjective well-being. * *p* < 0.05, ** *p* < 0.01, *** *p* < 0.001. Empty cells indicate that the respective variable was excluded from the sectoral model due to non-significance.

**Table 11:** Comparison of the impact of various factors on Subjective Well-Being (SWB), as determined by hierarchical binary regression models in different sectors when variable Health risks are excluded.

	Exp(b)				
Variable’s	Entire Sample N = 1323	Type1 (Public Administration) N = 217	Type2 (Healthcare) N = 325	Type3 (Energy) N = 432	Type4 (Pharma) N = 349
Social inclusion	5.107 ***	11.407 ***	5.357 ***	4.433 ***	4.174 ***
−Work intensity	0.513 ***	0.324 ***	0.569 **	0.429 ***	0.427 ***
−Health problems	0.245 ***	0.078 ***	0.303 ***	0.232 ***	0.357 ***
Autonomy	1.170 *			1.440 **	
−Age	1.046 **	1.043	1.040 *	1.071 ***	1.053 ***
Gender (men)		0.256 *	0.199 **		
Nagelkerke R Square	0.531	0.688	0.515	0.547	0.490
Hosmer–Lemeshow test *p*	0.002	0.894	0.357	0.145	0.077
AUC	0.872 ***	0.850 ***	0.865 ***	0.866 ***	0.867 ***

Note: A minus sign (–) before a variable’s indicates that higher values of this factor decrease the likelihood of high SWB (Exp(b) < 1). In this table, this applies to work intensity, health problems, and age. Factors without the minus sign (Exp(b) > 1) increase the likelihood of high SWB. * *p* < 0.05, ** *p* < 0.01, *** *p* < 0.001. Empty cells indicate that the respective variable was excluded from the sectoral model due to non-significance.

**Table 12:** Classification performance of logistic regression models (cut-off = 0.5).

Sector	Model	Overall Accuracy (%)	Sensitivity (%)	Specificity (%)
Whole sample	Control-only	59.4	63.2	55.3
	Full model	77.2	79.2	75.8
Public administration	Control-only	54.1	44.6	64.3
	Full model	74.5	67.1	82.5
Healthcare	Control-only	52.3	33.3	73.4
	Full model	74.5	72.9	76.3
Energy services	Control-only	57.6	65.5	50.0
	Full model	76.3	73.1	80.0
Pharmaceutical manufacturing	Control-only	55.5	49.0	63.4
	Full model	76.9	77.2	76.5

Note. n = 1323; Control-only model includes only demographic covariates (age, tenure, gender); Full model includes demographic covariates plus key psychosocial factors (social inclusion, work intensity, health problems, autonomy). Sensitivity = proportion of correctly classified High SWB cases; specificity = proportion of correctly classified Low SWB cases; overall accuracy = proportion of all correctly classified cases.

**Table 13:** Comparison of Binary and Ordinal Logistic Regression Models for Subjective Well-Being.

Variables	Binary Logistic (SPSS) OR [95% CI]	*p*	Ordinal Logistic (Rstudio) OR [95% CI]	*p*	Direction Same?
Age	1.04 [1.03–1.06]	<0.001	1.39 [1.23–1.57]	<0.001	Yes
Work experience	1.01 [0.99–1.03]	0.109	1.04 [0.92–1.16]	0.554	Yes
Gender (men)	0.81 [0.59–1.11]	0.205	0.79 [0.65–0.96]	0.021	Yes
Social inclusion	5.36 [4.22–6.81]	<0.001	3.02 [2.64–3.44]	<0.001	Yes
Professional growth	1.02 [0.95–1.11]	0.468	1.04 [0.99–1.09]	0.160	Yes
Work intensity	0.45 [0.37–0.54]	<0.001	0.56 [0.49–0.63]	<0.001	Yes
Health risks	1.28 [1.14–1.44]	<0.001	1.19 [1.11–1.28]	<0.001	Yes
Autonomy	1.24 [1.07–1.43]	0.003	1.10 [1.01–1.21]	0.034	Yes
Health problems	0.24 [0.18–0.33]	<0.001	0.52 [0.45–0.60]	<0.001	Yes
Metric					
Nagelkerke R^2^/Pseido R^2^	0.542		0.845		
Hosmer—Lemeshow *p*	<0.001		n/a (not applicable)		
AUC	0.813				
Classification accuracy	78.5%		73–75%		
Purpose	Main model		Sensitivity analysis		
			N = 1628		

## Data Availability

The data supporting the findings of this study are available from the corresponding author upon reasonable request.

## Supplementary Materials

The following supporting information can be downloaded at: https://www.mdpi.com/article/10.3390/bs16010157/s1.

## Abbreviations

SWB	Subjective Well-being
PSWB	Psychosocial Well-being
JD-R	Job Demands-Resources Model
MBI	Maslach Burnout Inventory
OR	Odds Ratio
OECD	Organisation for Economic Cooperation and Development
WHO	World Health Organization
RSU	Rigas Stradiņš university
SPSS	Statistical Package for the Social Sciences
CI	Confidence Interval
COVID-19	Coronavirus Disease 2019
N	Sample Size
SD	Standard Deviation
*p*	*p*-value (statistical)
