# Supplementary material for: Sectoral Differences in Psychosocial Well-Being: The Role of Work Environment Factors Across Public Administration, Healthcare, Pharmaceutical, and Energy Services
Source: Behav Sci (Basel). 2026 Jan 22;16(1):157. doi: 10.3390/bs16010157 (PMC12838197; doi:10.3390/bs16010157)
Supplement: Supplementary file 1 [file behavsci-16-00157-s001.zip › behavsci-4007908-supplementary.pdf]

Multidimensional scale of Psychosocial well-being for employed persons (MPSWEP)

Please indicate the extent to which you agree or disagree with the statements about your personal experience at work

Tick one answer per line

1<br>Strongly disagree	2<br>Disagree	3<br>Rather disagree	4<br>Rather agree	5<br>Agree	6<br>Strongly agree

1	My job offers good career opportunities	1	2	3	4	5	6

Please indicate how often you experience the following in the workplace

Tick one answer per line

1<br>At no time	2<br>Sometimes	3<br>Less than half the time	4<br>More than half the time	5<br>Most time	6<br>All the time

2	How often do you feel that you are living a fulfilling life?	1	2	3	4	5	6
3	How often do you feel involved in improving work processes in your organisation/team?	1	2	3	4	5	6
4	How often do you feel that you can influence decisions that are important for your work?	1	2	3	4	5	6
5	To what extent do you trust the top management of your organisation?	1	2	3	4	5	6
6	How often do you feel supported by your manager?	1	2	3	4	5	6
7	How often do you feel your life is in balance (work and personal time)?	1	2	3	4	5	6
8	How often are you exposed to chemicals at work?	1	2	3	4	5	6
9	How often are you exposed to noise at work?	1	2	3	4	5	6
10	How often do you feel emotionally drained by your work?	1	2	3	4	5	6
11	How often do you feel physically tired at the end of the working day?	1	2	3	4	5	6
12	How often can you choose your own working methods or change them as you see fit?	1	2	3	4	5	6

13	How often can you choose or change the order of your tasks?	1	2	3	4	5	6
14	How often do you have to work fast at high speed?	1	2	3	4	5	6
15	How often do you receive recognition or praise for a job well done?	1	2	3	4	5	6

Please indicate whether you have had any of the following experiences at work in the last year

Tick one answer per line

	1<br>Yes	2<br>No
16 In the past year, I have received training paid for or provided by my employer	1	2
17 In the last year, I have received training that improves my future job prospects	1	2
18 In the last year, I have received training that has improved my skills	1	2

Please indicate how often you experience the following in the workplace

Tick one answer per line

		1<br>At no time	2<br>Sometimes	3<br>Less than half the time	4<br>More than half the time	5<br>Most time	6<br>All the time
In the last two week...							
19	I was happy and in a good mood	1	2	3	4	5	6
20	I felt calm and relaxed	1	2	3	4	5	6
21	I felt energetic and active	1	2	3	4	5	6
22	After waking up I felt vivid and rested	1	2	3	4	5	6
23	My daily life was filled with things that interest me	1	2	3	4	5	6

Thank you very much!
